# Supplementary material for: Patient and caregiver perspectives of select non-communicable diseases in India: A scoping review
Source: PLoS One. 2024 Jan 5;19(1):e0296643. doi: 10.1371/journal.pone.0296643 (PMC10769076; doi:10.1371/journal.pone.0296643)
Supplement: S2 Table — (DOCX) [file pone.0296643.s002.docx]

**Supplementary Table S2. Data extraction form for study characteristics**

|  | **Items** | **Attributes** |
| --- | --- | --- |
| 1 | Author (First author) |  |
| 2 | Year of publication |  |
| 3 | Type of study design | 1. Quantitative – observational 2. Quantitative – interventions 3. Qualitative 4. Mixed methods 5. Comments – Open box |
| 4 | Objective(s) of the study |  |
| 5 | Study population | 1. Patients 2. Caregivers |
| 6 | Study settings | 1. Outpatient department 2. Inpatient/Ward 3. Emergency department 4. ICU 5. Community setting 6. Teleclinics/Mobile health care 7. Others |
| 7 | Study location | 1. Rural 2. Urban 3. Tribal 4. Not sure 5. Hospital-based |
| 8 | State(s) of India where the study was conducted | 1. Single State – text box 2. Multiregional text box 3. Representative across India |
| 9 | Level of healthcare setting | 1. Primary (Subcenter/Primary Health Centre/Community Health Centre/home based) 2. Secondary (Taluk/district) 3. Tertiary care (Medical college hospitals / higher specialty hospitals) 4. Not sure 5. Community based |
| 10 | Type of healthcare provider | 1. Government 2. Private/corporate 3. Charitable institution/not for profit 4. Others |
| 11 | Type of care being evaluated | 1. Screening (e.g., diabetic retinopathy/ screening for other disease among diabetics or cancer patients) 2. Diagnostic 3. Therapeutic/ chronic illness management 4. Palliative 5. Others – text box |
| 12 | Type of data collection | 1. Primary 2. Secondary 3. Others - text box |
| 13 | Sample size for patients | Text box |
| 14 | Sample size for caregivers | Text box |
| 15 | Age group of patients | Text box |
| 16 | Age group of caregivers | Text box |
| 17 | Sex distribution for patients | Males — numbers in textbox  Females — numbers in textbox |
| 18 | Sex distribution for caregivers | Males — numbers in textbox  Females — numbers in textbox |
| 19 | Outcomes mainly consist of | 1. Care experiences 2. Care preferences 3. Both |
| 20 | Type(s) of experiences reported based on  HCAHPS domains | 1. communication with doctors 2. communication with nurses 3. responsiveness of hospital staff, 4. pain management 5. communication about medicines 6. discharge information 7. cleanliness of the hospital environment 8. quietness of the hospital environment 9. transition of care 10. overall rating 11. recommend the facility |
| 21 | Any other outcomes related to care experiences | Text box |
| 22 | Outcomes related to care preferences that were measured | 1. Related to screening 2. Related to diagnosis of disease 3. Related to investigations/tests 4. Related to treatment/management decisions 5. Others |

**Supplementary Table S3. Data extraction form for key findings**

| **S. No.** | **Items** | **Outcomes** |
| --- | --- | --- |
| **1.** | Overall results  on experiences | 1. Mostly Positive  2. Mostly Negative  3. Mixed |
| **2** | Key findings related to experiences | Selected text from the article |
| **3** | Key findings related to preferences | Selected text from the article |
| **4** | Quotes |  |
| **5** | Remarks by reviewer |  |
