# Supplementary material for: Patient and caregiver perspectives of select non-communicable diseases in India: A scoping review
Source: PLoS One. 2024 Jan 5;19(1):e0296643. doi: 10.1371/journal.pone.0296643 (PMC10769076; doi:10.1371/journal.pone.0296643)
Supplement: S3 Table — (DOCX) [file pone.0296643.s003.docx]

**Supplementary Table S3. Data extraction form for key findings**

| **S. No.** | **Items** | **Outcomes** |
| --- | --- | --- |
| **1.** | Overall results  on experiences | 1. Mostly Positive  2. Mostly Negative  3. Mixed |
| **2** | Key findings related to experiences | Selected text from the article |
| **3** | Key findings related to preferences | Selected text from the article |
| **4** | Quotes |  |
| **5** | Remarks by reviewer |  |
