# Supplementary material for: Patient and caregiver perspectives of select non-communicable diseases in India: A scoping review
Source: PLoS One. 2024 Jan 5;19(1):e0296643. doi: 10.1371/journal.pone.0296643 (PMC10769076; doi:10.1371/journal.pone.0296643)
Supplement: S4 Table — (DOCX) [file pone.0296643.s004.docx]

**Supplementary Table S5. Descriptive characteristics of included diabetes-related studies (n = 49) in chronological order**

| **Author / year of publication/**  **PMID or URL** | **State** | **Study design, sample size, participants** | **Study settings, organisation** | **Type of care evaluated** | **Relevant outcomes** | **Strengths** | **Limitations** |
| --- | --- | --- | --- | --- | --- | --- | --- |
| Srinivas G, 2002, 12021154 | Tamil Nadu | Quantitative, Observational, 112 patients | Community settings (Rural) | Therapeutic | Hospital environment/ organizational aspects | Community based study on patient experiences | Limited to area catering to one primary health centre |
| Goghari MM, 2004, 14761111 | Gujarat | Qualitative, 1 patient case study | Outpatients (Private and government) | Therapeutic | Communication, Hospital environment, Overall satisfaction | In-depth patient and caregiver perspective | Limited sample size |
| Mehrotra R, 2004, 15638303 | Uttar Pradesh | Quantitative, Observational, 493 patients | Outpatients (Government) | Therapeutic | Complementary and alternate medicine (CAM) use with allopathy | Patient perspectives on CAM | No in-depth qualitative data |
| Peyrot M, 2005, 16176200 | Tamil Nadu | Quantitative, Observational,  105 type 1 and 299 type II DM patients in India | Hospital based (facility unsure), multi-country study | Therapeutic | Communication, not receiving psychological care | Robust methodology  Explored psychological issues | Sampling strategy likely to recruit more educated patients  Had used mixed face to face and telephone interviews c |
| Rani PK, 2006, 16638238 | Tamil Nadu | Mixed methods, 132 patients | Teleclinics (Private) | Screening | Communication, access to teleophthalmology | User experiences and preferences in teleophthalmology and hospital-based screening | No random sampling, findings cannot be generalized as based on single community in Tamil Nadu |
| Nagpal J, 2006, 17065665 | Delhi | Quantitative, Observational, 819 patients | Community settings (Urban) | Screening, Therapeutic | Overall satisfaction or rating | Patients recruited based on large survey, large sample size | Restricted to middle and high-income patients. No qualitative information |
| Kambar S, 2007, 18175402 | Karnataka | Quantitative, Observational,  532 family members | Caregivers  (Not for profit) | Therapeutic | Preferences related to management | Family members perspectives on management if they got diabetes, large sample size | No qualitative data, lack of generalisability |
| Varghese RT, 2007, <https://www.sciencedirect.com/science/article/abs/pii/S1871402107000410> | Kerala | Quantitative, Observational, 200 patients | Outpatients (Government) | Therapeutic | Hospital environment/ organizational aspects | Measured treatment satisfaction of outpatients | Minor outcome of health system related patient experiences |
| Kapur K, 2008, 18472496 | Tamil Nadu, Karnataka | Quantitative, Observational, 342 patients | Outpatients  (Private) | Therapeutic | Communication with dietician | Patients perceived barriers for dietary behaviour | Lack of in-depth qualitative information |
| Balaji A,  2011, 22315835 | Tamil Nadu | Quantitative, Observational, 323 patients | Outpatients (Private) | Therapeutic | Overall satisfaction or rating | Patients evaluated quality of care and preference of type of care reported. Mix of patients receiving treatment from private and public facilities | Findings based on single urban slum community and hence not generalisable |
| Shetty AS, 2011, 22616337 | Tamil Nadu | Quantitative, Interventional, 215 patients | Community settings (unsure) | Therapeutic | Communication, acceptability of SMS | Patients' acceptability of SMS | Minor outcome – patient preferences |
| Venkataraman K, 2012, 22048985 | Delhi | Quantitative, Observational, 507 patients | In-patients and outpatients (Private) | Screening, Therapeutic | Communication | Association of Treatment related factors with diabetic control | Minor outcome on patient experiences related to health system |
| Bhojani U, 2013, 24093885 | Karnataka | Qualitative, 16 patients | Community settings (Urban) | Therapeutic | Communication, Overall satisfaction or rating | In-depth qualitative information on urban slums patients | Limited sample size and limited geographical representation. |
| Bhalerao MS, 2013, 24093979 | Maharashtra | Quantitative, Observational, 200 patients | Outpatients (Government) | Therapeutic | Communication, Complementary and Alternate Medicine (CAM) use with allopathy | Treatment satisfaction with CAM | Minor outcome – patient experience |
| Gangwar SS, 2014, 25352870 | Uttar Pradesh | Quantitative, Interventional, 723 patients | Community (pharmacies) | Therapeutic | Communication (pharmacists in community pharmacies) | Drug related problem and psychological aspects in patients | Minor outcome of patient experiences. Mainly clinical outcomes |
| Sankar UV, 2015, 23417905 | Kerala | Mixed methods,346 patients | Community settings (Rural) | Therapeutic | Communication | Factors associated with drug adherence were explored | Minor outcome related to drug compliance |
| Debnath P, 2015, 25348958 | Karnataka | Quantitative, Observational, 36 patients | Outpatients (Private) | Therapeutic | CAM use with allopathy | treatment satisfaction of patients with diabetic ulcers treated with ayurvedic and insulin | Limited sample size  Limited scope |
| Kishore J, 2015, 26229715 | Delhi | Quantitative, Observational, Rural – 67, urban slums - 31 | Community settings (Rural and Urban) | Therapeutic | Hospital environment/ organizational aspects | Reasons for not taking treatment | Limited sample size, Rural area not representative of other parts of India.  Minor outcome of patient experiences. |
| Kakumani KV, 2016, 28405986 | Maharashtra | Quantitative, Observational, 70 patients | Community settings (rural) | Therapeutic | Overall satisfaction or rating | Reasons for non-compliance | Limited sample size, minor outcome related only to drug compliance |
| Madhu B, 2016, 27117890 | Karnataka | Quantitative, Observational, 104 patients | Community settings (Urban) | Therapeutic | Communication, Overall satisfaction or rating | Evaluated patient time spent with doctor | Minor outcome Quality of care was mostly clinical related |
| Hussain R, 2016, 27221678 | Kerala | Quantitative, Observational, 1538 patients | Community settings (Rural and Urban) | Screening, Therapeutic | Communication | Patient’s experiences and preferences about diabetic retinopathy explored | Minor outcome – patient experiences and preferences |
| Balasubramaniyan N, 2016, 27358634 | Tamil Nadu | Quantitative, Observational, 93 patients | Outpatients attached to primary care (Government) | Screening,  Therapeutic | Communication | Type of provider preference, communication regarding advice for eye screening | Minor outcome – patient experiences and preferences |
| George M, 2016, [A Study on Quality of Life Assessment among Patients with Type 2 Diabetes Mellitus in a Tertiary Care Hospital in Southern Kerala \| IJPPR (humanjournals.com)](https://ijppr.humanjournals.com/a-study-on-quality-of-life-assessment-among-patients-with-type-2-diabetes-mellitus-in-a-tertiary-care-hospital-in-southern-kerala/)  , | Kerala | Quantitative, Observational, 60 patients | Outpatients  (Private) | Therapeutic | Overall satisfaction or rating | Used a quality of life instrument for diabetes developed in India which included treatment satisfaction | Limited sample size  Minor outcome – patient experiences |
| Srinath KM, 2017, 28089167 | Karnataka | Quantitative,  Observational, 400 patients | Community settings (Rural) | Therapeutic | Communication | Large sample size, Random selection | Limited sample size; Minor outcome related to patient experiences |
| Kleinman NJ, 2017, 28328396 | Gujarat, Maharashtra,  Tamil Nadu | Quantitative, Interventional, 91 patients | Mobile health platform (Private) | Therapeutic | Communication, Overall satisfaction or rating | Patient experience with mobile application was evaluated | Patient satisfaction limited to medication adherence |
| Vishnu N, 2017, 29868217 | Kerala | Quantitative, Observational, 400 patients | Community settings (rural) | Therapeutic | CAM use | Large sample size and random selection, Experiences and preferences with CAM and allopathy | Based on one district in Kerala, hence not generalisable |
| Mentock SM, 2017, 28465150 | Karnataka, | Quantitative,  Observational 204 patients | Outpatients  (Private) | Therapeutic | Communication, Overall satisfaction or rating, Preferences about SMS | Patient doctor communication was explored well | Limited generalisability |
| Elias MA, 2017, 29527334 | Karnataka | Mixed methods, 663 diabetic patients | Community settings (unsure if rural/urban) | Therapeutic | Overall satisfaction or rating, unavailability of diagnostics | In depth exploration of patients experiences and highlights gaps in health care delivery in primary health care settings. Large sample size and well conducted study | Findings based on 3 talukas in a district in the state of Karnataka, so limited generalisability |
| Mendhe H, 2017, <https://www.ijcmph.com/index.php/ijcmph/article/view/1632> | Andhra Pradesh | Quantitative, Observational, 508 patients | In-patients and  outpatients (Private) | Therapeutic | Communication, Overall satisfaction or rating, Hospital environment/ organizational aspects | Patient perceived health system related barriers were explored | No qualitative data available, |
| Thakur A, 2018, 30294101 | Delhi | Quantitative, Observational, 150 patients | Outpatients (Government) | Therapeutic | Overall satisfaction or rating | Comparison of private versus government care experiences and reasons for choice | Limited to an urban resettlement in Delhi colony so poor generalisability |
| Rahman SAU, 2019, 29618311 | Andhra Pradesh | Quantitative, Observational, 430 patients | Outpatients (Government) | Therapeutic | Quality of care for monitoring sugars, Overall satisfaction or rating | Level of satisfaction for glucose monitoring system | Limited scope of patient experience assessment |
| Jayanna K, 2019, 30991978 | Karnataka | Qualitative (addressing current scoping review objectives), 30 patients | Community settings (urban) | Therapeutic | Organisational aspects | In-depth qualitative information on gaps in health care delivery | Limited sample size and generalisability |
| Acharya AS, 2019, 31299834 | Delhi | Quantitative, Observational, 200 patients | Outpatients (Private) | Therapeutic | Distance to facility, health care costs association with adherence to medication | Factors affecting medicine adherence | Limited generalisability  Limited to medicine adherence |
| Kotian SP, 2019, 32189656 | Maharashtra | Quantitative, Observational, 208 patients | Community settings (urban) | Therapeutic | Overall satisfaction or rating, reported with adherence | Factors affecting medicine adherence | Limited generalisability  Limited to medicine adherence |
| Tripathy JP, 2019, 31410044 | Delhi, Karnataka, Maharashtra | Qualitative (actual study design is mixed methods, qualitative focus on scoping review objectives, 67 patients | Out-patients (Government) | Therapeutic | Communication, Hospital environment/ organizational aspects | In depth exploration of challenges in health care delivery system at all levels for diabetes management | Limited generalisability |
| Kumar R, 2019, <https://www.jpmer.com/doi/JPMER/pdf/10.5005/jp-journals-10028-1314> | Chandigarh | Quantitative, Observational, 97 patients | Outpatients (Government) | Therapeutic | Overall satisfaction or rating with quality of life | Unique study to capture treatment satisfaction of type I diabetic patients, standard quality of life tool | Limited generalisability  No qualitative data which otherwise would have explained decreased treatment satisfaction |
| John R,  2019, 31391757 | Maharashtra | Quantitative, Observational, 153 patients | Inpatients  (Private) | Therapeutic | Overall treatment satisfaction or rating | Used modified and validated tool for quality of life in Indian diabetic patients in local (Marathi) language | Limited generalisability |
| Ramagiri R, 2020, 31937727 | Andhra Pradesh | Quantitative, interventional, 235 patients + qualitative – 11 diabetic patients + 2 FGDs (4 and 6 participants) | Community settings (urban) | Screening | Communication | Evaluated patients' feedback on preference for type of health education sources  Illustrative patient quotes on barriers and facilitators to Diabetic Retinopathy screening | Participants not randomly allocated to interventions, and outcomes not masked to evaluators |
| Anjana RM, 2020, 32522031 | Tamil Nadu | Quantitative, Observational, 2510 patients | Teleclinics / Mobile health care (Private) | Therapeutic | Telemedicine during COVID-19 pandemic | Large sample size,  Includes patient quotes on experiences or preferences regarding teleclinics | Limited generalisability  as  patients recruited from single tertiary centre |
| Raj JP, 2020,  32896089 | Karnataka | Quantitative, Interventional, 50 patients | In-patients (Private) | Therapeutic | Communication, barriers to adherence | Explored barriers to adherence | Limited sample size and generalisability |
| Basu S, 2020, 32670834 | Delhi | Quantitative, Observational, 375 patients | Outpatients (Government) | Therapeutic | Communication, Hospital environment/  organizational aspects | Large sample size, insights on communication and organizational issues | Limited generalisability, No random sampling |
| Basu S, 2020, 32950850 | Delhi | Quantitative, Observational, 339 patients | Outpatients (Government) | Therapeutic | Communication on oral health, Hospital environment/ organizational aspects | Unique study for treatment seeking practices for oral health in diabetics | Findings based on single centre, hence limited generalisability |
| Lohiya NN, 2020, 33180040 | Maharashtra | Quantitative, Observational, 67 mothers and children | Out-patients (Private) | Therapeutic | Overall satisfaction or rating | Unique study to capture type I diabetic patients and caregivers experiences | Limited sample size |
| Rana S, 2020, 33484987 | Telangana | Quantitative, Observational, 200 patients | Outpatients  (Not sure if private/government) | Therapeutic | Overall satisfaction or rating (patient centred chronic illness care assessment) | Standard tool was used to measure patient centred chronic care assessment. | Not very specific in reporting patient experience. Interpretation and results not clear. |
| Priya TK,  2020, 33457600 | Puducherry | Quantitative, Observational, 200 patients | Community settings (Urban) | Therapeutic | Overall satisfaction or rating | Included patients treated at both government and private facilities | Limited generalisability, convenience sample |
| Chaturvedi R, 2018, 29430413 | Gujarat | Quantitative, Observational, 200 patients | Outpatients (Government) | Therapeutic | Overall satisfaction or rating | Patient’s satisfaction reported related to type of drugs | Patients' satisfaction scope restricted to drugs  Limited generalisability |
| Deswal J, 2020, 32317460 | Chandigarh | Quantitative, Observational, 250 patients | Outpatients (Government) | Therapeutic | Overall satisfaction or rating | Patient’s satisfaction among patients with diabetic retinopathy, large sample size, | Treatment satisfaction restricted to disease status  Limited generalisability,patient subgroup selection unclear (not sure how many were there with no retinopathy as methods different from results) |
| Gupta J, 2020, 34760676 | Himachal Pradesh | Quantitative, Observational, 300 patients | In-patients and outpatients (Government) | Therapeutic | Overall satisfaction or rating | Factors associated with treatment satisfaction explored, standard questionnaire used | Limited generalizability |
| Nailwal D, 2021, 33708751 | Uttarakhand | Quantitative, Observational, 233 patients | Outpatients (Government) | Therapeutic | CAM use with allopathy | Objectively measured different reasons for choosing alternate medicines over allopathy | Findings based on single centre, limited generalizability,  Lack of qualitative data to support findings |
