# Supplementary material for: Patient and caregiver perspectives of select non-communicable diseases in India: A scoping review
Source: PLoS One. 2024 Jan 5;19(1):e0296643. doi: 10.1371/journal.pone.0296643 (PMC10769076; doi:10.1371/journal.pone.0296643)
Supplement: S5 Table — (DOCX) [file pone.0296643.s005.docx]

**Supplementary Table S6. Descriptive characteristics of included cancer-related studies (n = 46) in chronological order**

| **Author / year of publication/**  **PMID or URL** | **State** | **Study design, sample size, participants** | **Study settings, organisation** | **Type of care evaluated** | **Relevant outcomes** | **Strengths** | **Limitations** |
| --- | --- | --- | --- | --- | --- | --- | --- |
| Ramanakumar AV, 2005,  16101331 | Maharashtra | Qualitative,  52 patients | Cancer registry (population based) (urban) | Therapeutic | Treatment delay, survivorship experience | Patients identified from a population based registry, inductive coding (free listing) | Missing findings of interviewed not included for analysis, no illustrative quotes on treatment experience, |
| Nayak S, 2005, 16034053 | Odisha | Quantitative Interventional, 400 patients | Outpatients (Government) | Therapeutic | Communication changes with doctors after a targeted intervention | Detailed evaluation of communication | No control arm |
| Gupta D,2007, 17347507 | Delhi | Case report, one, caregiver | Outpatients (Government) | Therapeutic | Communication, preferences related to treatment | Detailed qualitative experiences | One caregiver’s, experiences of many prior providers |
| Mehrotra S, 2007, 17431687 | Karnataka | Qualitative, 20 caregivers | Hospital based  (setting not clear) (Government) | Therapeutic | Communication, responsiveness of staff | Reasons described for positive experience with hospital staff | Small study size |
| Gupta V, 2007, 17802985 | Uttar Pradesh | Mixed methods, 20 patients, 30 caregivers | Hospital based  (setting not clear) (Government) | Palliative | Preferences related to diagnosis and management | Details of palliative care preferences | No gender description of participants, |
| McGrath P, 2009, 20081722 | Kerala | Qualitative, 5 patients | Hospital based  (setting not clear) (Government) | Palliative | Communication, pain management | Well written qualitative methods and report | Small sample size and limited generalisability |
| Kannan G, 2011, 22044807 | Tamil Nadu | Quantitative, Observational, 32 patients | Outpatients  (Private) | Therapeutic | Communication, overall satisfaction or rating | Validated questionnaire with a component on healthcare | Care experiences only a minor component of QOL, small size |
| Chintamani, 2011, 22046495 | Delhi | Quantitative, Observational, 100 patients | Outpatients (Government) | Diagnostic, Therapeutic | Diagnostic delay, treatment initiation delay | Details of reasons for delays | Only breast cancer related |
| Akhtar M,  2011, 22293251 | Maharashtra | Quantitative, Observational, 71 patients | Outpatients (Government) | Diagnostic, Therapeutic | Diagnostic delay, treatment initiation delay | Details of reasons for delays | Only locally advanced breast cancer related |
| Chittem M, 2012, 22930492 | Andhra Pradesh | Quantitative, Observational, 329 patients | Outpatients  (Not for profit) | Therapeutic | Communication | Patients with various cancers represented | Experience only a minor outcome, opportunistic sampling |
| Ambulkar R, 2013, 23821135 | Maharashtra | Quantitative, Observational, 141 patients and their families | In patients (Government) | Therapeutic | Communication, information provided in the patient information leaflet, preferences for involvement in decisions | Clarity of results, detailed preferences of patients and caregivers | Caregiver number not given, only patients with thoracic cancers, |
| Pati S, 2013, 24211153 | Odisha | Mixed methods, 68 patients | In patients (Government) | Diagnostic,  Therapeutic | Diagnostic delay, treatment initiation delay | Mixed methods, delays quantified | Limited to patients reaching tertiary care |
| Laxmi S, 2013, 24455553 | Rajasthan | Quantitative,  Observational, 300 patients | Out-patients  (Not for profit) | Therapeutic | Preferences related to diagnosis and management | Details of information wanted, large sample size, various cancers | Limited tp preferences about information |
| Kaur N, 2014, 25494119 | Delhi | Mixed methods, 154 patients | Out-patients (Government) | Post treatment follow-up | Communication (information and rehabilitation needs for survivors) | Large sample of cancer survivors, open ended questions for needs assessment | Limited to breast cancer |
| Tiwari et al / 2015, [https://www.researchgate.net/publication/315890457_treatment_seeking_behaviour_and_referral_practices_a_study_of_the_cancer_patients_in_tertiary_care_hospitals_in_india/citation/download](https://www.researchgate.net/publication/315890457_TREATMENT_SEEKING_BEHAVIOUR_AND_REFERRAL_PRACTICES_A_STUDY_OF_THE_CANCER_PATIENTS_IN_TERTIARY_CARE_HOSPITALS_IN_INDIA/citation/download) | National | Quantitative Observational, 611 patients | Both in and Outpatients (Tertiary care) | Diagnostic,  Therapeutic | Treatment delay, referral processes, Hospital environment/ organizational aspects | Multiregional study, large sample size, detailed results | Non indexed, sampling methods not clear, results not applicable across all states which is not adequately reflected in discussion |
| Suriakala R, 2016, Shodhganga<http://hdl.handle.net/10603/294335> | Kerala | Quantitative, Observational,  459 patients | Outpatients  (Private) | Therapeutic | Communication, Hospital environment/ organizational aspects, overall satisfaction or rating | Multiple aspects evaluated along with overall rating | Tools not standard, poor generalisability, no random selection of participants, not peer reviewed |
| Nayak MG, 2016, Shodhganga  <http://hdl.handle.net/10603/169932> | Karnataka | Quantitative, Observational, 768 patients and their caregivers | Outpatients  (Private) | Therapeutic | Communication, pain management | Details of communication regarding pain, large sample size | Not peer reviewed, no control arm |
| Rao A, 2016, 27221852 | Karnataka | Quantitative, Observational, 150 patients, 150 caregivers | Inpatients and outpatients (Private) | Diagnostic | Preferences related to diagnosis | Different questionnaires for patients based on awareness of diagnosis, separate interviews for caregivers | Limited to disclosure of diagnosis, no demographic or medical details collected |
| Patil V, 2016, 27938994 | Maharashtra | Quantitative, Observational,  200 patients | Out-patients (Government) | Palliative | Preferences related to palliative treatment | Detailed preferences of terminally ill patients regarding palliation | Mainly males, only head and neck cancers |
| Nyblade L, 2017, 28768506 | Karnataka | Qualitative, 27 patients, 22 caregivers | Out-patients  (Private) | Stigma due to treatment | Stigma due to hospital visits for cancer treatment | Well written qualitative methods and illustrative quotes, figure | Patients with breast cancer only, most other results of cervical cancer are not from patients or caregivers |
| Deshmukh VL, 2017, <https://dx.doi.org/10.18203/2320-1770.ijrcog20174434> | Maharashtra | Quantitative, Observational, 100 patients | Out-patients (Government) | Diagnostic | Diagnosis missed by doctor | Group of patients seen in a tertiary centre, large rural representation | Limited to cervical cancer, delays in diagnosis only mentioned in one line in table |
| Yennurajalingam S, 2018, 29235415 | Maharashtra | Quantitative, Observational, 97 patients | Out-patients (Government) | Palliative | Communication, overall satisfaction or rating, preferences related to palliative treatment | Multi-country study, details of decision control preferences and satisfaction | Results from one tertiary centre in India |
| Bafna VS, 2018, 29368109 | Maharashtra | Quantitative, Observational, 70 caregivers | Out-patients  (Private) | Therapeutic | Overall satisfaction or rating | Validated WHO BREF score, caregivers of paediatric cancer patients | No specific details, only scores for domain with healthcare quality |
| Rath H, 2018, 29438925 | Odisha | Qualitative, 70 patients | Out-patients (Government) | Diagnostic,  Therapeutic | Communication, diagnostic delay, appointment delay, treatment delay | Well written qualitative methods and illustrative quotes, figures | Oral cancer only |
| Yanamandra U, 2018, 30241224 | Chandigarh | Quantitative, Observational, 333 patients | Out-patients (Government) | Therapeutic | Communication, hospital environment/ organizational aspects, | Large sample of patients, highlighted issues with tertiary care appointments | Only about issues with specific treatment for Chronic Myeloid Leukaemia |
| Shankar SD, 2018, 30241243 | Pondicherry | Quantitative, Observational, 133 patients | General surgery department (Government) | Therapeutic | Preferences related to treatment, information need | Various cancers represented, Information needs and preferences related to decisions for surgery | Interviewer bias of using a trainee physician to ask about preferences, mostly rural patients |
| Alam A, 2018, 30316108 | Uttar Pradesh | Quantitative, Observational, 87 caregivers | Out-patients and emergency (Government) | Therapeutic | Communication, reasons for treatment default | First study to explore reasons for treatment default in children with leukaemia, >90% follow up rate | Limited to children with leukaemia |
| Van Rijssen LB, 2019, 29261524 | Maharashtra | Quantitative Observational, 333 patients | In patients  (Government) | Therapeutic | Preferences related to patient reported outcome | Multi-country, Delphi approach for consensus | Pancreatic cancer only, results not specific to India |
| Sathiaraj E, 2019, 30260687 | Karnataka | Quantitative, Interventional, 160 patients | In patients  (Private) | Hospital services | Hospital environment (dietary management of cancer patients) | Trial of a patient experience improvement intervention | Limited to evaluating a food service model in one centre |
| Ghoshal A, 2019, 30757949 | Maharashtra | Quantitative, Observational, 150 patients | Out-patients (Government) | Palliative | Communication, preferences related to palliative care | Satisfaction with decision making and preferences studied, validated scale, clear methodology | Possible bias as interviews conducted by treating physicians |
| Harding R, 2019, 30837250 | Karnataka | Qualitative, 10 patients, 10 caregivers | Out-patients  (Not for profit), | Palliative | Communication, pain management, preferences related to palliative care | Explanatory model of experiences with terminal cancer, independent interviewers | Small numbers, mainly female patients, data from one private non-profit medical college |
| Hazarika M, 2019, 31030486 | Assam | Quantitative, Observational, 592 caregivers | Out-patients (Government) | Therapeutic | Treatment refusal and dropout due to poor access to health care for childhood cancer | Large sample size, reasons for abandonment of treatment explored | Large percentage not contacted, retrospective, recall bias |
| Gota V, 2019, 31397364 | Maharashtra | Quantitative,  Observational, 200 patients | Out-patients (Government) | Therapeutic | Communication, quality of process of informed consent of trials | Unique objective of experience in consent process, participants from various types of trials | Limited to experience with clinical trials in a single centre |
| Ghoshal A, 2019, 31770048 | Maharashtra | Quantitative, Observational, 250 patients, 250 caregivers | Out-patients (Government) | Therapeutic | Preferences related to communication needs including disclosure of disease status | Preferences of both patients and caregivers, large sample size, newly diagnosed patients, detailed analysis | Single tertiary centre study |
| Kumar A, 2019, 31870113 | Assam | Mixed methods, 284 patients, 10 caregivers | Out-patients (Government) | Therapeutic | Treatment delay | Conducted at a regional referral centre; many illustrative quotes, | Qualitative component small sample size, limited to breast cancer |
| Das K, 2020, 31044306 | Uttarakhand | Qualitative, 26 caregivers | Community settings (rural and urban) | Palliative | Communication, pain management, end of life experiences and preferences for place of care | Preferences, experiences, and regrets of parents studied after child’s death | Limited to childhood cancer, analysis is quantitative, parents reporting child’s stress |
| Ganesan S, 2020, 32592363 | Pondicherry | Mixed methods, 216 patients | Out-patients (Government) | Diagnostic | Diagnostic delay | Reasons for delay due to patient and health system factors | Limited to head and neck cancers |
| Ghosh J, 2020, 32552110 | West Bengal | Quantitative,  Observational, 302 patients | In patients and out-patients (Private) | Therapeutic | Preferences related to continuation of treatment during the pandemic | Large sample, variety of cancers | Limited to pandemic period, leading questions |
| Lokanatha D, 2020, 32593160 | Karnataka | Quantitative, Observational, 202 patients | Out-patients (Government) | Diagnostic | Referral delay, diagnostic delay, treatment initiation delay | Large sample size, details of delay from symptoms to treatment onset | Single centre, only gastric and oesophageal advanced cancers |
| Mahalakshmi S, 2020, 32334474 | Tamil Nadu | Qualitative, 1 patient | Community settings  (not mentioned if rural or urban) | Therapeutic | Communication, facilitators and barriers in screening | Quotes about experience of cancer survivor | Only one woman with cancer while others were from general population |
| Nidhi V, 2020, 33623300 | Karnataka | Quantitative, Observational, 148 caregivers | In patients (Private) | Therapeutic | Overall satisfaction or rating | 74% response rate, treatment satisfaction, preferences regarding disclosure of illness | Possible interviewer bias in assessing treatment satisfaction, limited to one private hospital |
| Panda J, 2020, <https://doi.org/10.1007/s40944-019-0358-2> | Odisha | Quantitative,  Observational, 122 patients | Out-patients (Government) | Diagnostic | Referral delay, diagnostic delay | Various types of delays experienced for cervical cancer care, three hospitals in state capital | Limited to cervical cancer patients |
| Rathore P, 2020, 33088114 | Delhi | Case report, one patient (child) and his caregiver | In patient  (Government) | Therapeutic | Communication, during COVID and cancer related hospitalisation | Illustrative of problems faced by cancer patients during the pandemic, perspectives of both caregiver and child patient | Limited to specific period of pandemic, not generalisable |
| Somanna SN, 2020, 31983180 | Karnataka | Quantitative Observational, 181 patients | Cancer registries (both hospital and population based) | Diagnostic,  Therapeutic | Referral delay, diagnostic delay, treatment initiation delay (breast cancer) | Wide source for recruiting patients, clear flow of patient inclusion | Limited to breast cancer experiences from one referral centre in a single state |
| Somanna SN, 2020, 33247688 | Karnataka | Quantitative,  Observational, 210 patients | Cancer registries (both hospital and population based) (urban) | Diagnostic,  Therapeutic | Diagnostic delay, treatment initiation delay (cervical cancer) | Detailed information on sources of delay | Limited to cervical cancer experiences from one referral centre in a single state |
| Kondeti AK, 2021, 34319033 | Andhra Pradesh | Quantitative, Observational, 211 caregivers | In patients (Government) | Palliative | Communication, pain management, overall satisfaction or rating, | Use of validated scale, clear flow of patient inclusion, various cancers | Limited to palliative care |
