# Supplementary material for: Patient and caregiver perspectives of select non-communicable diseases in India: A scoping review
Source: PLoS One. 2024 Jan 5;19(1):e0296643. doi: 10.1371/journal.pone.0296643 (PMC10769076; doi:10.1371/journal.pone.0296643)
Supplement: S1 File — (DOCX) [file pone.0296643.s006.docx]

**S1 File. Search strategy**

**1. Database: PubMed**

**Date of search: 27 November 2021**

**Filter**: Year - 2000 onwards, Language - English

Patients / caregivers [MESH / search terms] AND (Experiences [MESH / search terms] OR Preferences [MESH / search terms]) AND (Diabetes [MESH / search terms] OR Cancer [MESH / search terms] AND India

**Search results**: 1586

| **S. No** | **Search domains** | **Mesh terms** | **Search strategy (Keywords)** |
| --- | --- | --- | --- |
| 1 | Population:  Patients/caregivers | "Patients"[Mesh]) OR "Caregivers"[Mesh] OR "Child"[Mesh] OR "Family"[Mesh] | (((patient*[Title/Abstract] OR population*[Title/Abstract] OR community*[Title/Abstract] OR individual*[Title/Abstract] OR (child*[Title/Abstract]) OR care taker*[Title/Abstract] OR care giver*[Title/Abstract] OR care*[Title/Abstract] OR famil*[Title/Abstract] OR parent*[Title/Abstract]))) |
| 2 | Concepts | | |
| 2a | Experiences | (((("Physician-Patient Relations"[Mesh]) OR "Patient Acceptance of Health Care"[Mesh]) OR "Quality Assurance, Health Care"[Mesh]) | experience*[Title/Abstract] OR encounter*[Title/Abstract] OR care seeking[Title/Abstract] OR treatment seeking[Title/Abstract] OR cost*[Title/Abstract] OR expenditure*[Title/Abstract] OR feedback*[Title/Abstract] OR perception*[Title/Abstract] OR quality[Title/Abstract] OR assessment*[Title/Abstract] OR opinion*[Title/Abstract] OR physician-patient relation*[Title/Abstract] OR evaluation[Title/Abstract] OR challenge*[Title/Abstract] OR difficulties[Title/Abstract] OR problems[Title/Abstract]) |
| 2b | Preferences | ("Patient-Centered Care"[Mesh]) AND "Patient Satisfaction"[Mesh] | preference*[Title/Abstract] OR choice*[Title/Abstract] OR expectation*[Title/Abstract] OR value*[Title/Abstract] OR decision*[Title/Abstract] OR attitude*[Title/Abstract] OR need*[Title/Abstract] OR desire*[Title/Abstract])OR patient-centered care[Title/Abstract] OR shared- decision- making[Title/Abstract] OR collaborative-decision-making[Title/Abstract] OR medical decision-making[Title/Abstract] OR patient- autonomy[Title/Abstract] |
| 2c | Diabetes | (("Diabetes Mellitus"[Mesh]) | diabetes [Title/Abstract] |
| 2d | Cancer | "Neoplasms"[Mesh] | Cancer*[Title/Abstract] OR Carcinoma*[Title/Abstract] OR neoplasm*[Title/Abstract] OR malignant*[Title/Abstract] |
| 3 | Context | | |
| 4 | India | "India"[Mesh] | "India"[Title/Abstract] OR  "India"[Affiliation] OR  "India"[Place of Publication]) |
|  | SEARCH strategy with filter: Year - 2000 onwards, Language - English | | 1 (Mesh terms OR keywords) AND 2a (Mesh terms OR keywords) OR 2b (Mesh terms OR keywords) AND 3a(Mesh terms OR keywords) OR 3b (Mesh terms OR keywords) AND 4 (Mesh terms OR keywords) |

**Actual executed strategy PubMed search:**

(((("patient*"[Text Word] OR "caregiver*"[Text Word] OR "child" [MeSH Terms] " OR child*"[Title/Abstract] OR "famil*"[Text Word] OR "population*"[Title/Abstract] OR "communit*"[Title/Abstract] OR "caretaker*"[Title/Abstract] OR "caregiver*"[Title/Abstract] OR "parent*"[Title/Abstract]) AND ((("satisf*"[Title/Abstract] OR "experience*"[Title/Abstract] OR "encounter*"[Title/Abstract] OR "care seeking"[Title/Abstract] OR "treatment seeking"[Title/Abstract] OR "feedback*"[Title/Abstract] OR "perception*"[Title/Abstract] OR "Quality"[Title/Abstract]) AND "assessment*"[Title/Abstract]) OR "opinion*"[Title/Abstract] OR "Evaluation"[Title/Abstract] OR "challenge*"[Title/Abstract] OR "Difficulties"[Title/Abstract] OR "Problems"[Title/Abstract] OR "professional patient relation*"[Title/Abstract] OR "preference*"[Title/Abstract] OR "choice*"[Title/Abstract] OR "expectation*"[Title/Abstract] OR "value*"[Title/Abstract] OR "decision*"[Title/Abstract] OR "attitude*"[Title/Abstract] OR "need*"[Title/Abstract] OR "desire*"[Title/Abstract] OR "Patient-Centered Care"[Title/Abstract] OR "shared decision making"[Title/Abstract] OR "Collaborative-decision-making"[Title/Abstract] OR "medical decision making"[Title/Abstract] OR "patient autonomy"[Title/Abstract] OR "patient reported outcome*"[Title/Abstract] OR "Patient advocacy"[Title/Abstract] OR "Patient navigation"[Title/Abstract])) OR ("Patient Outcome Assessment"[MeSH Terms] OR "Patient navigation"[MeSH Terms] OR "Patient Education as Topic"[MeSH Terms] OR "Patient advocacy"[MeSH Terms] OR "Professional-Patient Relations"[MeSH Terms] OR "Patient Acceptance of Health Care"[MeSH Terms] OR "quality assurance, health care"[MeSH Terms] OR "Patient-Centered Care"[MeSH Terms] OR "Patient Satisfaction"[MeSH Terms])) AND ("diabetes mellitus"[MeSH Terms] OR "neoplasms"[MeSH Terms] OR ((("diabet*"[Title/Abstract] OR "Neoplasm"[Title/Abstract]) AND "or "cancer*"[Title/Abstract]) OR "malignant*"[Title/Abstract])) AND ("India"[MeSH Terms] OR "India"[Title/Abstract] OR "India"[Affiliation] OR "India"[Place of Publication]) AND 2000/01/01:2021/12/31[Date - Publication] AND "english"[Language] AND (study characteristics[pt] OR Epidemiologic Studies[mesh]) NOT review[pt])

**2. Database: Global Index Medicus**

**Date of search: 25 January 2022**

Using exact terms of PubMed, revealed no results, but adding only major terms revealed 117 results

(patient* OR caregiver* OR caretaker* ) AND (satisf* OR preference* OR feedback OR perception* OR attitude OR experience* OR quality of life) AND (diabet* OR cancer* OR neoplasm* OR palliative) AND india ) AND ( la:("en")) AND (year_cluster:[2000 TO 2021])

**3. Grey literature: Google Scholar**

**Date of search:** 8 February 2022

Using major terms, 100 article were retrieved from the first 10 pages and included in Level 1 screening.

**4. Grey literature: Shodhganga,** a database for master / doctoral thesis / dissertations [Shodhganga : a reservoir of Indian theses @ INFLIBNET](https://shodhganga.inflibnet.ac.in/)

**Date of search: 21 June 2022**

‘Title’ – This website does not search using combinations of words, but gives all available 2599 records in order of relevance so our search was limited to the first 10 articles. There was no option for abstract search. Keyword search did not provide any additional relevant records.

10 articles were screened for every line in the search strategy given below:

e.g. 10 for patient satisfaction, cancer

**Search 1 set**

1. Patient satisfaction diabetes

2. Patient experiences diabetes

3. Patient preferences diabetes

4. Patient expectations diabetes

5. Patient perceptions diabetes

No relevant hits

**Search 2 set**

6. Caregiver satisfaction diabetes

7. Caregiver experiences diabetes

8. Caregiver preferences diabetes

9. Caregiver expectations diabetes

10. Caregiver perceptions diabetes

No relevant hits

**Search 3 set**

11. Patient satisfaction cancer

12. Patient experiences cancer

13. Patient preferences cancer

14. Patient expectations cancer

15. Patient perceptions cancer

Found 2 relevant articles relevant

**Search 4 set**

16. Caregiver satisfaction cancer

17. Caregiver experiences cancer

18. Caregiver preferences cancer

19. Caregiver expectations cancer

20. Caregiver perceptions cancer

No relevant hits

**5. Hand search -** 19 articles, including 9 obtained by reviewing of references of included articles
