# Supplementary material for: Patient and caregiver perspectives of select non-communicable diseases in India: A scoping review
Source: PLoS One. 2024 Jan 5;19(1):e0296643. doi: 10.1371/journal.pone.0296643 (PMC10769076; doi:10.1371/journal.pone.0296643)
Supplement: Attachment — Submitted filename: Responsestoreviewers.docx [file pone.0296643.s007.docx]

Dear Editor and Reviewers

Thank you for your insightful comments which we believe has improved the readability of the manuscript.

Given below are our responses to reviewers’ questions. Changes in the manuscript have been placed as tracked changes.

| **Comment** | **Response** | **Changes made if any** |
| --- | --- | --- |
| 1. Is the manuscript technically sound, and do the data support the conclusions?  Reviewer #1: Partly  Reviewer #2: Yes | We hope that the specific changes made in response to other comments done would have addressed Reviewer 1’s rating. We also broke up long sentences to improve readability. | |
| 2. Has the statistical analysis been performed appropriately and rigorously?  Reviewer #1: N/A  Reviewer #2: N/A | N/A | |
| 3. Have the authors made all data underlying the findings in their manuscript fully available?  Reviewer #1: Yes  Reviewer #2: Yes | No change asked for | |
| 4. Is the manuscript presented in an intelligible fashion and written in standard English?  Reviewer #1: No  Reviewer #2: Yes | We have revised the language and format; please let us know if you have any further specific suggestions for improvement | Grammatical corrections done using grammarly.com (throughout the manuscript, highlighted as tracked changes) and formatting changes were done throughout, according to PLOS One requirements which was also pointed out to us by the Editor.  References are also placed in square brackets and heading formatted according to PLOS One guidelines.  Tables also have now been placed in the text as indicated in the guidelines.  Double spacing done; headings formatted. |
| Reviewer #1: The findings will enhance understanding of the current healthcare services in mentioned area. It will help to improved on the area that need to be improvised. Further enhancement on the format and language need to be done to increase the quality of the paper |  |  |
| Reviewer #2:  1. Please give appropriate rationale for selecting the two non communicable diseases, viz. Diabetes and Cancer, and not including Hypertension for the review, which is a risk factor for cardiovascular deaths in India. | Yes, we have deliberately chosen only two kinds of common NCDs as it would have taken the review even longer to do and lengthier to read if we included more conditions. Both DM and cancer are common NCDs, and also represent diseases with multiple complications and aspects of treatment with two different types of care pathways, so we chose those as examples of NCDs. We have now added further explanations in limitations based on this concern, acknowledging the importance of hypertension and other common NCDs. | Introduction page 4, para 3  Discussion, page 22, para 3 |
| 2. Appreciate the authors that they have mentioned how the keywords for search was selected. Kindly mention the keywords and MeSH terms used in various permutations for conducting the literature search. | The various combinations of search terms are provided in Supplementary File S1 for all the databases used.  This was too long for the methods section and hence is in a supplementary file table. | Mentioned in methods that search terms are given in supplementary File S1: page 6, lines 1-2.  Supplementary File S1 gives  both the MeSH terms and keyword permutations.  We hope the supplementary file is accessible to the reviewers. |
| 3. Please include a table reflecting the general study characteristics including the study setting, year of study and strengths and limitations of each study included in the scoping review, which would enable the readers to better put the findings and also observe any time trend specific changes in patient and caregiver perspective, since the infrastructure,  services under NPCDCS (Now NPNCD) and guidelines have been dynamic and evolving. | Since there are nearly a 100 studies this table is given as a set of two supplementary tables S4 and S5. We have added the missing components suggested by Reviewer 2.  We have arranged in chronological order so that readers can view these in table format, and also see changes over time, although such comparative assessment is difficult due to wide heterogeneity in methods and results between studies.  We have added comments in results and discussion on differences in study outcomes over time. | Supplementary Tables S4 and S5 (added more descriptives, strengths and limitations)  Results:  Page 9, para 1,2  Page 15, para 2  Page 17, para 1  Discussion: page 24, para 2 |
| 4. It would be good to see geographically stratified analysis done for patient satisfaction or at least include a write up regarding this in the discussion section, since in India, health is a state subject and wide patient and caregiver differentials are observed in different settings. | We have done a sub analysis as suggested by looking at % with predominantly negative experiences (either overall poor satisfaction or other negative experiences related to communication, delays etc.), based on geographical regions (as already classified in results under ‘characteristics of included studies and key outcomes’). This sub analysis has now been mentioned in results and discussion. | Results: page 7, para 2  (classification of regions as given in Table 1 and Results page 9, last para)  Discussion page 25, last para.  Page 26, para 1 |
