# Supplementary material for: Patient and caregiver perspectives of select non-communicable diseases in India: A scoping review
Source: PLoS One. 2024 Jan 5;19(1):e0296643. doi: 10.1371/journal.pone.0296643 (PMC10769076; doi:10.1371/journal.pone.0296643)
Supplement: Attachment — Submitted filename: ResponsestoreviewersNov2023.docx [file pone.0296643.s008.docx]

Dear Editor and Reviewers

Thank you for your comments and feedback regarding the readability of the manuscript.

Given below are our responses to reviewer’s questions. Changes in the manuscript have been placed as tracked changes.

| **Comment** | **Response** | **Changes made if any** |
| --- | --- | --- |
| Reviewer #3:   Introduction:  The introduction section of the article is very limited and need to be elobarated more,  I didn't observe the chronological sequence of presenting the data related to the subject matter,  some paragraphs within this section are supposed to be within the method section. | We have added a few more lines in the intro.  **The current intro is in the following sequence:**   - Responsiveness of health systems and India’s performance in the global health quality assessment study, - Need for assessing experiences and preferences, - Lack of systems for assessing quality of health facilities in India unlike some other countries, - Burden of NCDs and the lack of reviews on NCD care, - Rationale of choosing diabetes and cancer, - Research questions (objectives)   We felt that all of the above should remain in Intro and not methods.  We would welcome suggestions from the Editor/Reviewer 3 about ***which specific paragraphs currently in introduction*** we could move to methods. | Intro (page 3) para 1, lines 3,4, 6-9  Page 4: para 2, lines 10-12 |
| Citation errors observed.  Methods: Citation errors within the method. | We thank the reviewer for identifying the citation errors, for which we apologize. Citation errors have been checked and corrected.  One reference **(Ref 1)** had a wrong title while others had formatting errors.  There are no retracted articles in the references (response to Editor). | Reference errors corrected throughout. |
| Concepts and context: Not explained. | In response to the reviewer’s comments, we have rearranged the section on definitions of outcomes (which are the concept definitions) to come immediately after the PCC para and hope that this has improved the readability.  Context: modifications made | Rearranged methods with operational definitions to come in the PCC section  Page 6, line 11 (context) |
| Discussion:  Talked about more about study limitations in here which not suppose to be, the comparison with other studies is not well organized.  Mentioned conclussions and recommendations in you discussion section. | As there are multiple outcomes and studies are very heterogenous, comparison to other studies is given within each outcome heading and not as a single separate paragraph in the Discussion.  As other points were already discussed under each outcome heading, we have mainly discussed limitations and implications of findings in the discussion.  Recommendations are mentioned as a separate paragraph, but perhaps previously was not clear to the reader as there was no heading (added now).  We have added one more recommendation related to public reporting of quality assessment.  Conclusions are in the last para.  (We apologize that we were not sure what this comment meant as it was not clear if the comment refers to the fact that we have already mentioned recommendations and conclusions or have not mentioned them). | Discussion:  Recommendations- page 25, last line and page 26, first line |
| English: The language of the article need to be higher standard. | We have revised the language again and also asked a native English speaker to review and extensively corrected based on suggestions given. | Tracked changes throughout the manuscript.  Grammatical corrections previously done using grammarly.com |
